# Contralesional Cortical Structural Reorganization Contributes to Motor Recovery after Sub-Cortical Stroke: A Longitudinal Voxel-Based Morphometry Study

**DOI:** 10.3389/fnhum.2016.00393

**Published:** 2016-08-03

**Authors:** Jianxin Cai, Qiling Ji, Ruiqiang Xin, Dianping Zhang, Xu Na, Ruchen Peng, Kuncheng Li

**Affiliations:** ^1^Department of Radiology, Beijing Luhe Hospital, Capital Medical UniversityBeijing, China; ^2^Department of Neurology, Beijing Luhe Hospital, Capital Medical UniversityBeijing, China; ^3^Department of Radiology, Xuanwu Hospital, Capital Medical UniversityBeijing, China

**Keywords:** ischemic stroke, sub-cortical, structural MRI, voxel-based morphometry, motor recovery, plasticity, reorganization, gray matter volume

## Abstract

Although changes in brain gray matter after stroke have been identified in some neuroimaging studies, lesion heterogeneity and individual variability make the detection of potential neuronal reorganization difficult. This study attempted to investigate the potential structural cortical reorganization after sub-cortical stroke using a longitudinal voxel-based gray matter volume (GMV) analysis. Eleven right-handed patients with first-onset, subcortical, ischemic infarctions involving the basal ganglia regions underwent structural magnetic resonance imaging in addition to National Institutes of Health Stroke Scale (NIHSS) and Motricity Index (MI) assessments in the acute (<5 days) and chronic stages (1 year later). The GMVs were calculated and compared between the two stages using nonparametric permutation paired *t*-tests. Moreover, the Spearman correlations between the GMV changes and clinical recoveries were analyzed. Compared with the acute stage, significant decreases in GMV were observed in the ipsilesional (IL) precentral gyrus (PreCG), paracentral gyrus (ParaCG), and contralesional (CL) cerebellar lobule VII in the chronic stage. Additionally, significant increases in GMV were found in the CL orbitofrontal cortex (OFC) and middle (MFG) and inferior frontal gyri (IFG). Furthermore, severe GMV atrophy in the IL PreCG predicted poorer clinical recovery, and greater GMV increases in the CL OFG and MFG predicted better clinical recovery. Our findings suggest that structural reorganization of the CL “cognitive” cortices might contribute to motor recovery after sub-cortical stroke.

## Introduction

Brain damage after ischemic stroke can cause a greater variety of functional deficits. These deficits can be caused by direct damage to the cortices or fiber tracts, For example, injuries to the right inferior parietal lobe, superior temporal gyrus and inferior frontal gyrus (IFG) are frequently associated with neglect (Corbetta and Shulman, 2011), and lesions to the corticospinal tract (CST) can cause hemiplegia (Lo et al., 2010). In addition to the direct damage caused by lesions, indirect atrophy of lesion-related remote cortices has also been reported (Rowan et al., 2007; Gauthier et al., 2012; Fan et al., 2013; Zhang et al., 2014; Cheng et al., 2015). For example, a recent research has demonstrated that cortical atrophy in remote cortices is also correlated with the magnitude of residual motor deficits in chronic sub-cortical stroke patients (Gauthier et al., 2012). In addition to the evidence of secondary cortical atrophy, many early studies also reported secondary degeneration of remote white matter tracts after damage to the motor pathway due to sub-cortical stroke (Thomalla et al., 2004, 2005; Liang et al., 2008; Yu et al., 2009; Rüber et al., 2012), and the severity of the degeneration predicts poor motor recovery (Yu et al., 2009; Lindenberg et al., 2010). These findings indicate that the secondary neurodegeneration of the motor pathways might be responsible for the atrophy of remote cortical regions and might consequently influence motor performance.

Although secondary structural impairment of remote cortex has frequently been reported, the structural plasticity of the remaining cortex after stroke has yet to be fully clarified. In a review of previous neuroimaging literature that focused on the cortical changes after stroke, we found that the majority of studies adopted either a retrospective or cross-sectional design, and heterogeneities in lesion location and duration were frequently present (Schormann and Kraemer, 2003; Kraemer et al., 2004; Schaechter et al., 2006; Rowan et al., 2007; Stebbins et al., 2008; Gauthier et al., 2012). Individual variability in cross-sectional studies might mask subtle changes in the cortex or induce some false positive results, and lesion heterogeneity increases the complexity of interpreting the underlying neuronal mechanism. In a recent study of longitudinal changes in cortical thickness 3 months after sub-cortical stroke (Brodtmann et al., 2012), the authors found thickening of the contralesional (CL) cortices; however, these authors did not find any atrophy of the ipsilesional (IL) cortices. In another similar longitudinal study, Cheng et al. (2015) failed to identify any changes in cortical thickness in the CL lesion-connected or lesion-unconnected cortices, although they observed a strong decrease in the cortical thickness of the IL lesion-connected cortex. The discrepancy between the two studies may have been caused by the following factors: (1) the relative shorter follow-up duration (3 months after stroke); (2) the insensitivity of the region-of-interest (ROI) analysis method; and (3) the constraint of the cortical thickness in completely characterizing cortical atrophy and plasticity without accounting for changes in cortical surface area.

In this study, we recruited a subset of motor-deficit stroke patients with first onset, subcortical ischemic infractions that involved the basal ganglia regions. In contrast to early longitudinal studies (Brodtmann et al., 2012; Cheng et al., 2015), we used a whole-brain voxel-based morphometry (VBM) method to identify the potential changes in gray matter volume (GMV) after stroke. Moreover, the follow-up duration in the present study was extended to 1 year. Because the GMV contains information about both cortical thickness and cortical surface area, any changes in these two metrics can be reflected by the GMV. Based on the features of the VBM method and the longer follow-up duration, we hoped to identify both atrophy and augment in the GMV of the remote cortices after 1 years. Specifically, based on early studies that revealed secondary cortical atrophy of the IL cortices and its association with clinical performance, we hypothesized that the GMVs of the IL motor-related cortices would be decreased after 1 year, and the severity of the atrophy of these cortices would be associated with clinical recovery. Because early studies also demonstrated wide-spread of functional reorganization of multiple brain network (Wang et al., 2010, 2014; Rehme et al., 2012), we also hoped to observed increases in the GMVs of the remaining cortices and significant association between GMV increases and clinical recovery.

## Materials and Methods

### Subjects

The inclusion criteria were as follows: (1) first-onset stroke with a duration <5 days; (2) manifest motor deficits; (3) single ischemic infarct lesion involving the basal ganglia regions as confirmed by T2 weighted (T2W) imaging and diffusion weighted (DW) imaging; and (4) right-handed before the stroke. The exclusion criteria were as follows: (1) recurrence during the follow-up; (2) any other brain abnormalities, including tumors, vascular malformation, cerebral hemorrhage, etc.; (3) total occlusion of middle cerebral artery or internal carotid artery; and (4) patients with severe white-matter T2W hyperintensities were also excluded because many early studies have revealed that white matter hyper-intensities on T2W images are closely correlated with cortical atrophy (Rossi et al., 2006; Wen et al., 2006) and which would also confound the gray/white matter segmentation during VBM. We initially recruited 18 patients among whom two cases (1 female) suffered recurrent brain infarction, and five (3 female) cases were lost during the follow-up. Finally, 11 male stroke patients treated from 2010 to 2012 (mean age: 48.9 ± 6.5 years, range: 40–58 years old) were enrolled in the study since 2010 until 2012. Neurological examinations, including the National Institutes of Health Stroke Scale (NIHSS) and the Motricity Index (MI) of the involved upper and lower extremities (Collin and Wade, 1990), were performed at the acute (within 5 days after onset) and chronic (1 year later) stages. This experiment was approved by the Ethical Committee of the Beijing Luhe Hospital of Capital Medical University, and written informed consent was obtained from each subject before the study.

### MR Examinations

High resolution 3D T1-weighted (T1W) images were acquired with a brain volume (BRAVO) sequence on a 1.5T GE-HD MR scanner during the acute and chronic phase. The scanning parameters included: repetition time/echo time/inversion time = 8.0/1.7/450 ms, flip angle = 10°, 176 contiguous sagittal slices with a thickness of 1.2 mm, field of view = 25.6 × 25.6 cm, and matrix = 256 × 256. All subjects were instructed to remain motionless during the scanning. All images were free of obvious artifacts such as motion blurring and aliasing. Routine T2W and DW images were acquired to localize the lesions. The obtained 3D data were subjected to the preprocessing described below.

### Lesion Identification

**Figure 1:**
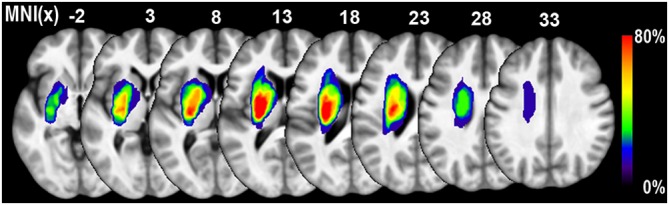
**Lesion probability map of 11 patients with sub-cortical stroke.** The color bar represents the probability of lesions. The lesion of each patients was defined based on the T2 weighted (T2W) images acquired in the acute stage.

We used the T2W images at the acute stage to identify the ischemic lesions. The contour of the sub-cortical lesion of each patient was drawn on graphic software MRIcron^1^ and was saved as binary images. Lesion size of each subject was calculated by summing all the non-zero voxels of the binary lesion images. Then individual T2W images and binary lesion images were registered to the 3D T1W images space using rigid body transformation. Next, the individual binary lesion images were normalized into the Montreal Neurological Institute (MNI) space using the Jacobian determinants that derived from the VBM steps. All the normalized lesion maps were overlapped to generate a lesion probabilistic map (Figure 1). A group lesion mask with lesion probability higher than zero was also generated, which was accordingly used as the excluded mask during the voxel-wise statistics.

### Voxel Based Morphometry

VBM was analyzed using a VBM8 toolbox implemented in SPM8^2^. Before VBM, the structural images with right infarct lesion (four cases) were right-to-left flipped; thus the left hemisphere represents the IL side, and the right represents the CL side. The main steps of the VBM included the following: segmentation, normalization, and smoothing. First, the native 3D structural images for each patient in the acute stage were rigidly registered with those of the chronic stage. Then the images were segmented into gray matter, white matter and CSF probability maps, representing the intensities of each tissue. A binary lesion mask of each subject was introduced during segmentation to prevent misclassification of the gray matter. During the normalization step, the native gray matter intensity (GMI) maps of the acute stage were first affinely registered into the SPM default tissue probability template. Next, the affined GMI maps were further nonlinear normalized into the MNI space using the Diffeomorphic Anatomical Registration Through Exponentiated Lie Algebra (DARTEL) algorithm (Ashburner, 2007). Subsequently, the chronic GMI maps were also transformed into the MNI space using the Jacobian determinant derived from normalization steps of the acute stage. The GMV map was obtained by multiplying the GMI map by the non-linear determinants while ignoring the linear affine determinates, which, in effect, represented the relative GMVs corrected for individual brain sizes^3^. Finally, the GMV maps were smoothed with a full-width at half-maximum (FWHM) kernel of 8 × 8 × 8 mm.

### Statistical Analysis

To identify the longitudinal changes in the GMVs of the remote cortices after sub-cortical stroke, we applied a nonparametric permutation-based paired *t*-test (Winkler et al., 2014) which was implemented in FSL software version 5.0^4^. This statistics algorithm randomized the samples between the acute and chronic stage within each subject, and this process was iterated 2^11^ = 2048 non-repeated times to generated a null distribution. In contrast to parametric statistics, the permutation test does not require a normality hypothesis and can exactly control for false positives when the exchangeability assumption of the samples is satisfied. To diminish white matter contamination, we restricted the permutation test within a gray matter mask, and the voxels containing infarct lesions were also excluded. The multiple compassions were corrected using a threshold-free cluster enhancement (TFCE) method correcting for family-wise error (FWE; *P* < 0.05). We also performed the permutation-based paired *t*-test to compare the differences in NIHSS and MI scores between the chronic and acute stage (*P* < 0.05, uncorrected).

To clarify whether the GMV changes between the acute and chronic stroke stages were consistent across the subjects, the average GMV of each ROI during each stage for each subject was extracted based on the voxel-wise permutation test. Specifically, the clusters with voxels that exhibited GMV changes (*P* < 0.05, TFCE FWE correction) and were within a 9 mm-radius ball at peak centering were defined as the ROIs.

We also performed ROI-wise correlation analyses to clarify whether the changes in GMV would be associated with the changes in the clinical variables (NIHSS and MI scores). After correcting for lesion volume, nonparametric Spearman correlation analyses were performed to test the associations between the changes in GMV and the clinical variables (NIHSS and MI; *P* < 0.05, Bonferroni correction).

## Results

### General Information

The demographic data for the recruited stoke patients were provided in Table 1. All of the patients were male. Seven and four cases suffered ischemic infarction in the left and right sub-cortical regions, respectively. As illustrated in Figure 1, the lesion locations were restricted to the basal ganglia regions. And the average lesion volume was 14.00 ± 13.42 ml (range: 2.88–45.77 ml). The average durations from stroke onset to MRI scanning was 2.0 ± 1.7 days in acute stage and 55.4 ± 7.1 weeks in chronic stage, respectively. The NIHSS scores were 11.6 ± 4.31 and 4.27 ± 3.63, and the MI scores were 15.6 ± 14.0 and 64.1 ± 29.1 during the acute and chronic stage, respectively. Significant functional recovery was detected via the application of permutation-based paired *t*-test to both the NIHSS (*P* < 0.001) and MI (*P* < 0.001) measurements.

**Table 1:** **Demographic information of recruited stroke patients**.

Patients	Age (years)	Gender	Lesion location	Lesion volume (ml)	NIHSS		MI	
					Acute	Chronic	Acute	Chronic
STR01	53	Male	Right Put, Caud, IC	3.78	18	2	0	95
STR02	43	Male	Left Caud, Insula, CR	8.67	14	0	13	95
STR03	51	Male	Left Put, Caud, CR	25.46	18	10	0	24
STR04	55	Male	Left Put, Caud, IC	8.61	7	5	19	48
STR05	54	Male	Left Put, Caud	13.18	13	5	7	56
STR06	50	Male	Left Caud, CR	2.88	10	10	22	37
STR07	58	Male	Right Put, Caud	7.91	8	5	7	24
STR08	45	Male	Right Caud, CR	6.02	7	1	34	99
STR09	38	Male	Left Put, Caud, IC, CR	27.41	8	2	7	67
STR10	40	Male	Left Put, IC	4.32	16	0	18	96
STR11	51	Male	Right Caud, Insula, CR	45.77	9	7	45	65

*Note: NIHSS, National Institutes of Health Stroke Scale; MI, Motricity Index; Put, putamen; Caud, caudate nucleus; IC, internal capsule; CR, corona radiata*.

### Gray Matter Volume Changes After Sub-Cortical Stroke

**Figure 2:**
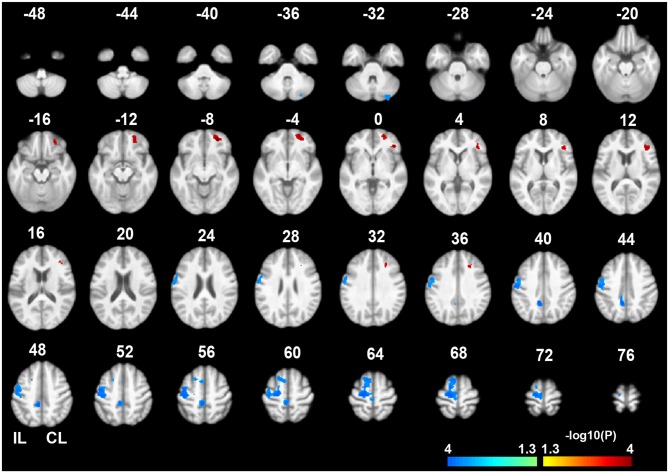
**Gray matter volume (GMV) differences between the chronic and acute stages of sub-cortical stroke.** The color bar represents the negative logarithmic P values of a permutation-based paired *t*-test (*q* < 0.05, FWE corrected). Warm colors indicates brain regions with increased GMVs in the chronic stage relative to the acute stage, and cool colors indicate brain regions with decreased GMVs. The IL PreCG, paracentral gyrus (ParaCG), and CL CBVII exhibited significant GMV atrophy in the chronic stage. In contrast, significant increases in GMV were found in the CL OFC, MFG and IFG. Abbreviations: CL, contralesional; GMV, gray matter volume; FWE, family-wise error; IL, ipsilesional; PrecCG, precentral gyrus; ParaCG, paracentral gyrus; CBVII, cerebellum lobule VII; OFC, orbital frontal cortex; MFG, middle frontal gyrus; IFG, inferior frontal gyrus.

**Figure 3:**
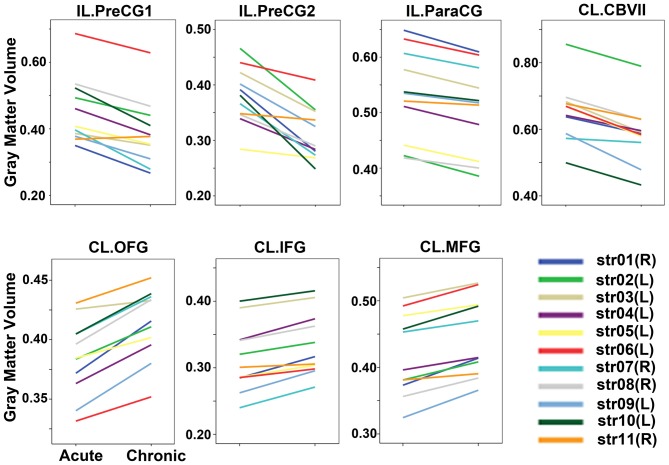
**GMVs of each patient in the acute and chronic stages.** The GMVs of each ROI in each stage are drawn for each subject. The changes in GMV were highly consistent across subjects. Abbreviations: CL, contralesional; GMV, gray matter volume; IL, ipsilesional; PrecCG, precentral gyrus; ParaCG, paracentral gyrus; CBVII, cerebellum lobule VII; OFC, orbital frontal cortex; MFG, middle frontal gyrus; ROI, region of interest; IFG, inferior frontal gyrus.

Voxel-wise permutation-based paired *t*-tests of the GMVs between the chronic and acute stages of stroke demonstrated significant decreases in GMVs in the IL precentral gyrus (PreCG), paracentral gyrus (ParaCG), and CL cerebellum lobule VII Crus 1 and Crus 2 (CBVII) regions. In contrast, significant increases in the GMVs was observed in the CL orbitofrontal cortex (OFC), IFG, and middle frontal gyrus (MFG; Figure 2, *P* < 0.05, FWE corrected). ROI-based individual analysis revealed that the changes in GMV were highly consistent across subjects (Figure 3).

### Correlations Between the Changes in GMV and the Clinical Variables

**Figure 4:**
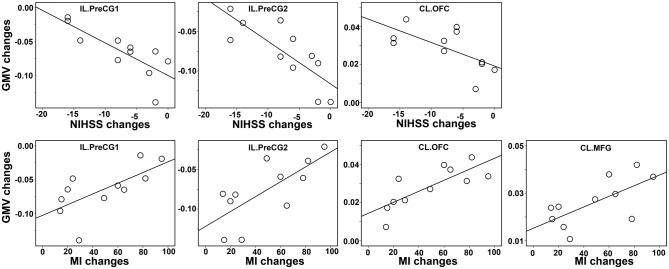
**Scatter plot of the associations between the changes in GMVs and clinical variables.** The associations were based on nonparametric Spearman correlation analyses after controlling for lesion volume effects. Only significant correlations (*P* < 0.05, uncorrected) are illustrated in this plot. The detailed statistical results are provided in Table 2. Abbreviations: CL, contralesional; GMV, gray matter volume; IL, ipsilesional; PrecCG, precentral gyrus; OFC, orbital frontal cortex; MFG, middle frontal gyrus; NIHSS, National Institutes of Health Stroke Scale; MI, Motricity Index.

The Spearman correlation coefficients for the relationships between GMV changes and the clinical variables (NIHSS and MI) were provided in Table 2 and Figure 4. After correcting for lesion volume, the severity of atrophy in the GMV at IL PreCG predicted poorer clinical recovery (for NIHSS: *P* < 0.05, Bonferroni corrected; for MI: *P* < 0.05. uncorrected). While a greater GMV increase in the CL OFC predicted better clinical recovery (for NIHSS, *P* = 0.04, uncorrected; for MI: *P* = 0.002, Bonferroni corrected), and a greater GMV increase in the CL MFG predicted better MI recovery (*P* = 0.047, uncorrected).

**Table 2:** **Correlation between changes in GMV and clinical variables in the stroke patients**.

	NIHSS		MI	
	*r*	*P*	*r*	*p*
**IL.ParaCG**	0.216	0.524	0.164	0.631
**IL.PreCG1**	**−0.826**	0.002*	**0.718**	**0.013**
**IL.PreCG2**	**−0.798**	0.003*	**0.618**	**0.043**
**CL.CBVII**	−0.372	0.261	0.209	0.537
**CL.IFG**	−0.482	0.134	0.455	0.160
**CL.MFG**	−0.427	0.191	**0.609**	**0.047**
**CL.OFC**	**−0.624**	**0.040**	**0.818**	0.002*

Note: *Indicates significant Spearman correlation survived under Bonferroni correction (P < 0.05). CL, contralesional; IL, ipsilesional; NIHSS, National Institutes of Health Stroke Scale; MI, Motricity Index; GMV, gray matter volume; PrecCG, precentral gyrus; ParaCG, paracentral gyrus; CBVII, cerebellum lobule VII; OFC, orbital frontal cortex; MFG, middle frontal gyrus; IFG, inferior frontal gyrus.

## Discussion

In accordance with recent VBM studies (Yabuta and Callaway, 1998; Gauthier et al., 2012), we found GMV decreases after stroke in some intact remote cortices that were directly or indirectly connected with the basal ganglia regions through cortical projection fibers (e.g., the CST, cortical-striatal-thalamic pathway, and cerebro-ponto-cerebellar circuit). Focal damage in these pathways can lead to dysfunction in neuroanatomically related remote brain regions soon after ischemia. Region specific diaschisis in the CL cerebellum after stroke has been identified (Lin et al., 2009), and might be correlated with clinical outcomes (Szilágyi et al., 2012). Similarly, secondary neurodegeneration has been identified in earlier studies using diffusion tensor imaging (DTI) in sub-cortical stroke patients (Liang et al., 2008; Crofts et al., 2011). Finally, diaschisis and degeneration can induce secondary atrophy in remote cortices (Yabuta and Callaway, 1998; Cheng et al., 2015) as observed in present study. These findings indicate that neurodegenerative atrophy in these “healthy” areas might partially account for permanent motor deficits. Except for the CBVII, we didn’t observe any significant atrophy in other “cognitive” regions that also have projections through the basal ganglia regions, and similar results have been reported in previous studies (Yabuta and Callaway, 1998; Rowan et al., 2007; Gauthier et al., 2012). One possible reason for this findings is that the motor function was predominantly involved in our patients, and thus the most significant atrophies occurred in motor-related areas.

So far, only a few studies identified GMV increases after stroke. Efficacious constraint-induced movement therapy has been found to induce increases in GMV in the bilateral sensorimotor cortices and hippocampi of chronic stroke patients (Woodhead et al., 2011). Fan et al. (2013) found increases in GMV ipsilesionally in the medial cingulate cortex (MCC), lingual gyrus and cerebellar lobe, contralesionally in the hippocampus and bilaterally in the precuneus via the monitoring the dynamic structural evolution after sub-cortical stroke (Yabuta and Callaway, 1998). Moreover, Brodtmann et al. (2012) observed thickening of the CL cortices 3 month after sub-cortical stroke. Animal experiments have demonstrated that the GMV increases in spared cortices may result from rehabilitation-induced increases in dendritic arborization and synaptic density (Holtmaat and Svoboda, 2009). In present study, we identified GMV increase in CL hemishpere, which was contradicted to a recent study that failed to identify any structural plasticity after sub-cortical stroke (Cheng et al., 2015). The positive findings of CL cortical structural plasticity in present study ascribe from: (1) longer follow-up interval: (1 year in present study vs. 3 month by Cheng et al., 2015); (2) different structural measurements (GMV in present study vs. cortical thickness by Cheng et al., 2015); and (3) whole brain voxel-wise search strategies. It should be noted that our findings with increased GMV were mainly located in CL prefrontal “cognitive” regions (MFG, OFC and IFG) rather than motor-related cortices (such as the premotor cortex and supplementary area; Yabuta and Callaway, 1998; Schaechter et al., 2006; Woodhead et al., 2011). In healthy subjects, the dorsal lateral prefrontal cortex is closely associated with attention (Corbetta et al., 2008), execution control (Goldman-Rakic, 1987) and motor learning (Tomassini et al., 2011), and the OFC is involved in motivational, emotional and social behavior (Rolls, 2004) and the integration for executive motor control (Cavada et al., 2000). Although the exact mechanism is far from clear, prior studies have demonstrated that cognitive abilities are particularly important for the recovery of motor function (Leung et al., 2010), and cognitive strategy-based interventions have beneficial effects on the recovery of motor function in stroke patients (Cirstea et al., 2006). Furthermore, a recent study demonstrated that a motor learning task can invoke greater activity in the dorsolateral prefrontal cortices in stroke patients with hemiplegia (Meehan et al., 2011). In this study, we also found that greater increases in the GMVs of these “cognitive” regions predicted better motor performances, indicating that the structural reorganization of these “cognitive” cortices may play an important role in motor recovery after stroke.

Several limitations should be noted. First, the sample size is relatively small, and the statistical power is thus low. To increase the statistical power, we right-left flipped the structure data of patients with right infarct lesions. Although this method was frequently applied in early stroke studies (Gauthier et al., 2008; Sharma et al., 2009; Li et al., 2013), it may be inappropriate for evaluating the brain areas with functional lateralization, such as the language and spatial processing areas. Because the number of patients with left-hemisphere lesions (seven cases) was much greater than that of the patients with right-side lesions (four cases), our findings (especially the increased GMV in the CL prefrontal cortex) may have primarily been contributed by patients with left-side infarctions. To clarify whether the patients with right-side infractions would exhibit patterns of change similar to those of the patients with left-side infarctions, we plotted the GMV changes subject-by-subject (Figure 3). We found that among the four right-side infraction patients (patient IDs: str01, str07, str08, and str11), with the exception of case str11, who manifested relatively weak GMV changes (but without the reverse changes), the remaining three patients exhibited change amplitudes comparable to those of the cases with left lesions. Thus, the laterality of the GMV changes may not have been significant in the present study. Of course, this assumption should be verified in the future with larger sample sizes. Second, we did not evaluate the cognitive functions at any stage in these patients, thus, the possibility that the augmentations in the GMVs reflected the compensatory plasticities of specific cognitive functions cannot be excluded.

In conclusion, by applying a longitudinal and lesion-restricted approach, we observed secondary neurodegeneration in remote cortices after sub-cortical stroke. We have also provided evidence of cortical plasticity in healthy cognitive-related cortices that might contribute to motor recovery. Further studies of adaptive plasticity in these remote areas may be important for developing and evaluating strategies for therapeutic intervention.

## Author Contributions

KL, JC and RP designed the experiment. JC, XN, QJ, DZ and RX performed the experiments. JC and QJ analyzed the data. JC, KL and RP drafted and revised the manuscript. All authors discussed the results.

## Conflict of Interest Statement

The authors declare that the research was conducted in the absence of any commercial or financial relationships that could be construed as a potential conflict of interest.

## Footnotes

^1^www.sph.sc.edu/comd/rorden/mricron/

^2^http://www.fil.ion.ucl.ac.uk/spm

^3^http://dbm.neuro.uni-jena.de/vbm8/VBM8-Manual.pdf

^4^http://fsl.fmrib.ox.ac.uk/fsl/

## References

[B1] Ashburner J. (2007). A fast diffeomorphic image registration algorithm. Neuroimage 38, 95–113. 10.1016/j.neuroimage.2007.07.007 17761438

[B2] Brodtmann A. Pardoe H. Li Q. Lichter R. Ostergaard L. Cumming T. (2012). Changes in regional brain volume three months after stroke. J. Neurol. Sci. 322, 122–128. 10.1016/j.jns.2012.07.019 22858417

[B3] Cavada C. Company T. Tejedor J. Cruz-Rizzolo R. J. Reinoso-Suarez F. (2000). The anatomical connections of the macaque monkey orbitofrontal cortex. A review. Cereb. Cortex 10, 220–242. 10.1093/cercor/10.3.220 10731218

[B4] Cheng B. Schulz R. Bönstrup M. Hummel F. C. Sedlacik J. Fiehler J.. (2015). Structural plasticity of remote cortical brain regions is determined by connectivity to the primary lesion in subcortical stroke. J. Cereb. Blood Flow Metab. 35, 1507–1514. 10.1038/jcbfm.2015.74 25920957 PMC4640340

[B5] Cirstea C. M. Ptito A. Levin M. F. (2006). Feedback and cognition in arm motor skill reacquisition after stroke. Stroke 37, 1237–1242. 10.1161/01.str.0000217417.89347.63 16601218

[B6] Collin C. Wade D. (1990). Assessing motor impairment after stroke: a pilot reliability study. J. Neurol. Neurosurg. Psychiatry 53, 576–579. 10.1136/jnnp.53.7.576 2391521 PMC488133

[B8] Corbetta M. Patel G. Shulman G. L. (2008). The reorienting system of the human brain: from environment to theory of mind. Neuron 58, 306–324. 10.1016/j.neuron.2008.04.017 18466742 PMC2441869

[B7] Corbetta M. Shulman G. L. (2011). Spatial neglect and attention networks. Annu. Rev. Neurosci. 34, 569–599. 10.1146/annurev-neuro-061010-113731 21692662 PMC3790661

[B9] Crofts J. J. Higham D. J. Bosnell R. Jbabdi S. Matthews P. M. Behrens T. E.. (2011). Network analysis detects changes in the contralesional hemisphere following stroke. Neuroimage 54, 161–169. 10.1016/j.neuroimage.2010.08.032 20728543 PMC3677803

[B10] Fan F. Zhu C. Chen H. Qin W. Ji X. Wang L.. (2013). Dynamic brain structural changes after left hemisphere subcortical stroke. Hum. Brain Mapp. 34, 1872–1881. 10.1002/hbm.22034 22431281 PMC6869928

[B11] Gauthier L. V. Taub E. Mark V. W. Barghi A. Uswatte G. (2012). Atrophy of spared gray matter tissue predicts poorer motor recovery and rehabilitation response in chronic stroke. Stroke 43, 453–457. 10.1161/STROKEAHA.111.633255 22096036 PMC3265680

[B12] Gauthier L. V. Taub E. Perkins C. Ortmann M. Mark V. W. Uswatte G. (2008). Remodeling the brain: plastic structural brain changes produced by different motor therapies after stroke. Stroke 39, 1520–1525. 10.1161/STROKEAHA.107.502229 18323492 PMC2574634

[B13] Goldman-Rakic P. S. (1987). Motor control function of the prefrontal cortex. Ciba Found. Symp. 132, 187–200. 10.1002/9780470513545.ch12 3322715

[B14] Holtmaat A. Svoboda K. (2009). Experience-dependent structural synaptic plasticity in the mammalian brain. Nat. Rev. Neurosci. 10, 647–658. 10.1038/nrn2699 19693029

[B15] Kraemer M. Schormann T. Hagemann G. Qi B. Witte O. W. Seitz R. J. (2004). Delayed shrinkage of the brain after ischemic stroke: preliminary observations with voxel-guided morphometry. J. Neuroimaging 14, 265–272. 10.1177/1051228404264950 15228769

[B16] Leung A. W. Cheng S. K. Mak A. K. Leung K. K. Li L. S. Lee T. M. (2010). Functional gain in hemorrhagic stroke patients is predicted by functional level and cognitive abilities measured at hospital admission. NeuroRehabilitation 27, 351–358. 10.3233/NRE-2010-0619 21160125

[B17] Li W. Han T. Qin W. Zhang J. Liu H. Li Y.. (2013). Altered functional connectivity of cognitive-related cerebellar subregions in well-recovered stroke patients. Neural Plast. 2013:452439. 10.1155/2013/452439 23862075 PMC3703724

[B18] Liang Z. Zeng J. Zhang C. Liu S. Ling X. Xu A.. (2008). Longitudinal investigations on the anterograde and retrograde degeneration in the pyramidal tract following pontine infarction with diffusion tensor imaging. Cerebrovasc. Dis. 25, 209–216. 10.1159/000113858 18216462

[B19] Lin D. D. Kleinman J. T. Wityk R. J. Gottesman R. F. Hillis A. E. Lee A. W.. (2009). Crossed cerebellar diaschisis in acute stroke detected by dynamic susceptibility contrast MR perfusion imaging. Am. J. Neuroradiol. 30, 710–715. 10.3174/ajnr.a1435 19193758 PMC2944923

[B20] Lindenberg R. Renga V. Zhu L. L. Betzler F. Alsop D. Schlaug G. (2010). Structural integrity of corticospinal motor fibers predicts motor impairment in chronic stroke. Neurology 74, 280–287. 10.1212/WNL.0b013e3181ccc6d9 20101033 PMC3122304

[B21] Lo R. Gitelman D. Levy R. Hulvershorn J. Parrish T. (2010). Identification of critical areas for motor function recovery in chronic stroke subjects using voxel-based lesion symptom mapping. Neuroimage 49, 9–18. 10.1016/j.neuroimage.2009.08.044 19716427

[B22] Meehan S. K. Randhawa B. Wessel B. Boyd L. A. (2011). Implicit sequence-specific motor learning after subcortical stroke is associated with increased prefrontal brain activations: an fMRI study. Hum. Brain Mapp. 32, 290–303. 10.1002/hbm.21019 20725908 PMC3010500

[B23] Rehme A. K. Eickhoff S. B. Rottschy C. Fink G. R. Grefkes C. (2012). Activation likelihood estimation meta-analysis of motor-related neural activity after stroke. Neuroimage 59, 2771–2782. 10.1016/j.neuroimage.2011.10.023 22023742

[B24] Rolls E. T. (2004). The functions of the orbitofrontal cortex. Brain Cogn. 55, 11–29. 10.1016/S0278-2626(03)00277-X 15134840

[B25] Rossi R. Boccardi M. Sabattoli F. Galluzzi S. Alaimo G. Testa C.. (2006). Topographic correspondence between white matter hyperintensities and brain atrophy. J. Neurol. 253, 919–927. 10.1007/s00415-006-0133-z 16502217

[B26] Rowan A. Vargha-Khadem F. Calamante F. Tournier J. D. Kirkham F. J. Chong W. K.. (2007). Cortical abnormalities and language function in young patients with basal ganglia stroke. Neuroimage 36, 431–440. 10.1016/j.neuroimage.2007.02.051 17462915

[B27] Rüber T. Schlaug G. Lindenberg R. (2012). Compensatory role of the cortico-rubro-spinal tract in motor recovery after stroke. Neurology 79, 515–522. 10.1212/wnl.0b013e31826356e8 22843266 PMC3413760

[B28] Schaechter J. D. Moore C. I. Connell B. D. Rosen B. R. Dijkhuizen R. M. (2006). Structural and functional plasticity in the somatosensory cortex of chronic stroke patients. Brain 129, 2722–2733. 10.1093/brain/awl214 16921177

[B29] Schormann T. Kraemer M. (2003). Voxel-guided morphometry (“VGM”) and application to stroke. IEEE Trans. Med. Imaging 22, 62–74. 10.1109/tmi.2002.806571 12703760

[B30] Sharma N. Simmons L. H. Jones P. S. Day D. J. Carpenter T. A. Pomeroy V. M.. (2009). Motor imagery after subcortical stroke: a functional magnetic resonance imaging study. Stroke 40, 1315–1324. 10.1161/strokeaha.108.525766 19182071

[B31] Stebbins G. T. Nyenhuis D. L. Wang C. Cox J. L. Freels S. Bangen K.. (2008). Gray matter atrophy in patients with ischemic stroke with cognitive impairment. Stroke 39, 785–793. 10.1161/STROKEAHA.107.507392 18258824

[B32] Szilágyi G. Vas A. Kerényi L. Nagy Z. Csiba L. Gulyás B. (2012). Correlation between crossed cerebellar diaschisis and clinical neurological scales. Acta Neurol. Scand. 125, 373–381. 10.1111/j.1600-0404.2011.01576.x 21781057

[B33] Thomalla G. Glauche V. Koch M. A. Beaulieu C. Weiller C. Röther J. (2004). Diffusion tensor imaging detects early Wallerian degeneration of the pyramidal tract after ischemic stroke. Neuroimage 22, 1767–1774. 10.1016/j.neuroimage.2004.03.041 15275932

[B34] Thomalla G. Glauche V. Weiller C. Röther J. (2005). Time course of wallerian degeneration after ischaemic stroke revealed by diffusion tensor imaging. J. Neurol. Neurosurg. Psychiatry 76, 266–268. 10.1136/jnnp.2004.046375 15654048 PMC1739511

[B35] Tomassini V. Jbabdi S. Kincses Z. T. Bosnell R. Douaud G. Pozzilli C.. (2011). Structural and functional bases for individual differences in motor learning. Hum. Brain Mapp. 32, 494–508. 10.1002/hbm.21037 20533562 PMC3674543

[B36] Wang C. Qin W. Zhang J. Tian T. Li Y. Meng L.. (2014). Altered functional organization within and between resting-state networks in chronic subcortical infarction. J. Cereb. Blood Flow Metab. 34, 597–605. 10.1038/jcbfm.2013.238 24398939 PMC3982082

[B37] Wang L. Yu C. Chen H. Qin W. He Y. Fan F.. (2010). Dynamic functional reorganization of the motor execution network after stroke. Brain 133, 1224–1238. 10.1093/brain/awq043 20354002

[B38] Wen W. Sachdev P. S. Chen X. Anstey K. (2006). Gray matter reduction is correlated with white matter hyperintensity volume: a voxel-based morphometric study in a large epidemiological sample. Neuroimage 29, 1031–1039. 10.1016/j.neuroimage.2005.08.057 16253521

[B39] Winkler A. M. Ridgway G. R. Webster M. A. Smith S. M. Nichols T. E. (2014). Permutation inference for the general linear model. Neuroimage 92, 381–397. 10.1016/j.neuroimage.2014.01.060 24530839 PMC4010955

[B40] Woodhead Z. V. Brownsett S. L. Dhanjal N. S. Beckmann C. Wise R. J. (2011). The visual word form system in context. J. Neurosci. 31, 193–199. 10.1523/jneurosci.2705-10.2011 21209204 PMC6622763

[B41] Yabuta N. H. Callaway E. M. (1998). Functional streams and local connections of layer 4C neurons in primary visual cortex of the macaque monkey. J. Neurosci. 18, 9489–9499. 9801386 10.1523/JNEUROSCI.18-22-09489.1998 PMC6792868

[B42] Yu C. Zhu C. Zhang Y. Chen H. Qin W. Wang M.. (2009). A longitudinal diffusion tensor imaging study on Wallerian degeneration of corticospinal tract after motor pathway stroke. Neuroimage 47, 451–458. 10.1016/j.neuroimage.2009.04.066 19409500

[B43] Zhang J. Meng L. Qin W. Liu N. Shi F. D. Yu C. (2014). Structural damage and functional reorganization in ipsilesional m1 in well-recovered patients with subcortical stroke. Stroke 45, 788–793. 10.1161/STROKEAHA.113.003425 24496396

